# Comparative miRNA expression profile analysis of porcine ovarian follicles: new insights into the initiation mechanism of follicular atresia

**DOI:** 10.3389/fgene.2023.1338411

**Published:** 2023-12-20

**Authors:** Jingge Liu, Caibo Ning, Jinbi Zhang, Shiyong Xu, Jiege Wu, Chenyu Tao, Fanhua Ma, Qing Chen, Zengxiang Pan

**Affiliations:** ^1^ College of Animal Science and Food Engineering, Jinling Institute of Technology, Nanjing, China; ^2^ College of Animal Science and Technology, Nanjing Agriculture University, Nanjing, China; ^3^ College of Animal Science and Technology, Hebei Agricultural University, Baoding, China

**Keywords:** miRNAs, pig ovary, follicle atresia initiation, function analysis, sequencing

## Abstract

Follicular atresia occurs in every stage of ovarian development, which is relevant to female fertility. In the past decade, increasing studies have confirmed that miRNAs, a class of short non-coding RNAs, play an important role in follicular atresia by post-transcription regulation of their target genes. However, the function of miRNAs on follicular atresia initiation is unknown. In the present study, high-throughput small RNA sequencing was performed to analyze differential miRNA expression profiles between healthy (HF) follicles and early atretic (EAF) follicles. A total of 237 conserved miRNA were detected, and the miR-143 is the highest expressed in follicles. Meanwhile, we also found wide sequence variations (isomiRs) in porcine ovarian miRNA, including in 5′un-translation region, core seed sequences and 3′untranslation region. Furthermore, we identified 22 differentially expressed miRNAs in EAF groups compared to HF group, of which 3 miRNAs were upregulated, as well as 19 miRNAs were downregulated, and then the RT-PCR was performed to validate these profiles. The target genes of these differentially expressed miRNAs were predicted by using miRwalk, miRDB, and Targetscan database, respectively. Moreover, the gene ontology and KEGG pathway enrichment established that the regulating functions and signaling pathways of these miRNAs contribute to follicular atresia initiation and cell fate. In conclusion, this study provides new insights into the changes of miRNAs in early atretic follicles to demonstrate their molecular regulation in ovarian follicular atretic initiation.

## 1 Introduction

The developing ovarian follicles will undergo four stages: continuous recruitment of primordial follicles, cyclic recruitment of small antral follicles, selection of potentially ovulatory follicles, and selection of dominant follicles (Jiang et al., 2003). In this process, follicles eventually face two kinds of fate: atresia or ovulation. Approximately 99% of follicles will undergo atresia and be eliminated in the follicular pool (DeFelici, 1997; Kaipia and Hsueh, 1997; Chu et al., 2018; Zhou et al., 2019). It is known that excessive follicular atresia will result in low fertility efficiency and premature ovarian failure (Liang et al., 2018). Recently, numerous pieces of evidence reveal that follicular atresia was induced by granulosa cell apoptosis, which occurred much earlier than morphological changes of atresia such as pyknotic nuclei, vessel disruption and GC layer fragment (Hussein, 2005; Rodgers and Irving-Rodgers, 2010; Zhang et al., 2018a). It also has confirmed that gonadotropins (e.g., FSH and LH) (Shen et al., 2017), steroid hormones (e.g., estrogen and progesterone) (Dierschke et al., 1994), cell growth factor (e.g., transforming growth factor and insulin-like growth factor) (Du et al., 2016), endothelial growth factor (e.g., CTGF and VEGF), oxidative damage responder (e.g., FoxO1 and NRF2) play essential roles in follicular atresia via controlling the activation of proapoptotic molecules (Lin et al., 2014; Shen et al., 2014; Shen et al., 2018). Meanwhile, a large number of key genes and key pathways involved in follicular atresia were identified by cDNA microarrays or RNA sequencing (Bonnet et al., 2008; Hatzirodos et al., 2014a; Hatzirodos et al., 2014b; Terenina et al., 2017; Zhang et al., 2018a). However, considering the gene expression level was regulated by multiple factors, including DNA methylation, histone modification and non-coding RNA regulation, there are limitations to just building gene-gene networks, and it is still unknown which factors lead to this marker gene expression change during follicular atresia.

MicroRNAs (miRNAs) are a kind of short non-coding RNA molecule (∼22 nucleotides; nt) that suppress gene expression post-transcriptionally by binding to the 3ʹ-UTR region to mediate mRNA degradation or translational repression (Li et al., 2018; Bofill-De Ros et al., 2019; Louer et al., 2019). It has been estimated that nearly all mRNA transcripts contain miRNA response elements (MREs), and more than 60% of human genes have been identified as regulated by miRNA (Friedman et al., 2009; Zhang et al., 2019a). Within the miRNA-induced silencing complex (miRISC), miRNAs have been identified to play an important role in multiple diseases (e.g., cancer, endometriosis and inflammation) and biological processes (embryonic development, muscle growth and brain function) (Piwecka et al., 2017). In porcine atretic follicles, accumulating miRNAs were identified to modulate GCs apoptosis through their target genes and signaling pathways, such as miR-26b, miR-181b, miR-10a-5p and miR-1275 (Liu et al., 2014; Liu et al., 2016; Liu et al., 2018; Guo et al., 2019). However, it should be noted that individual miRNAs can regulate multiple genes, and one gene is targeted by multiple miRNAs, indicating that the previous study, which only focused on one miRNA and its one target, was limited. Although several studies have built the miRNA target network in atretic follicles and apoptotic GCs (Donadeu et al., 2017; Du et al., 2019; Sohel et al., 2019), it is still unknown whether the miRNA regulatory network is involved in the initiation of follicular atresia. In the present study, we focused on miRNA profiling and their putative targets in early atretic follicles to provide new insights to elucidate initiation mechanisms of follicular atresia.

## 2 Materials and methods

### 2.1 Sample description

Duroc-Yorkshire-Landrace ovaries were obtained from three 700-day-old multiparous sows at a local slaughterhouse and subsequently transferred to the laboratory in physiological saline at 30°C–35°C. Antral follicles of 3–5 mm in diameter were selected to isolate morphologically healthy follicles (H) and early atretic (EA) with small forceps. Generally, healthy follicles are rounded with evenly distributed blood vessels; they have a fixed and visible cumulus–oocyte complex with clear follicular fluid; they are pink or yellow in appearance (Li et al., 2020a; Lin et al., 2012). Early atretic follicles may still have a visible COC but with gaps in membrane granulosa cells and turbid follicular fluid (Li et al., 2020a; Lin et al., 2012). Subsequently, the follicles were subjected to size measurement and then torn apart to obtain the mural GCs by scraping the follicular wall. GCs were immediately restored at −80°C for RNA-seq and qRT-PCR.

### 2.2 Small RNA sequencing and data analysis

GCs were collected from healthy and early atretic antral follicles of 3–5 mm in diameter. Then, RNA was isolated using miRVana total RNA isolation (ThermoFisher, Waltham, Massachusetts, United States) for sRNA-seq. Small RNA-seq libraries were randomly prepared from 400 ng of RNA using the NEXTFLEX Small RNA-Seq Kit v3 (PerkinElmer, Waltham, Massachusetts, United States). Sequencing of high-quality miRNA was performed on the Illumina HiSeq 2,500 (Illumina, San Diego, CA, United States). A detailed description of alignment, mapping and normalization was processed as previously described (Liu et al., 2019a). DEseq2 was used to analyze the differentially expressed (DE) miRNAs. MiRNAs with |foldchange|≥1.5 and *p* < 0.05 were identified as DE miRNAs. TargetFinder Software (https://github.com/carringtonlab/TargetFinder), TargetScan (www.targetscan.org), miRDB (www.miRDB.org), miRTarBase (www.miRTarBase.mbc.nctu.edu.tw) databases and TarBase (http://diana.cslab.ece.ntua.gr/tarbase/) were used to predict the targets of the differentially expressed miRNA. GO enrichment (http://www.geneontology.org) and KEGG pathway (http://www.genome.jp/kegg/pathway.html) analysis was performed as previously described (Liu et al., 2019a). The SeqBuster-mirAligner v.1.2.2 (Pantano et al., 2010) allowed the identification of isomiRs by performing a survey on miRNA sequence variability in the samples (analyzed in triplicate), applying default parameters.

### 2.3 qRT-PCR validation of the sequencing data

Quantitative reverse-transcription polymerase chain reaction (qRT-PCR) was used to validate the sequencing data. Samples used for total RNA isolation were described in the sample description. The Mir-X microRNAs First Strand Synthesis Kit (TaKaRa, Dalian, China) synthesized the complementary DNA (cDNA). Primers used here are listed in Table 1. The qRT-PCR was conducted using SYBR Green Master Mix (Vazyme, Nanjing, China) on the ABI QuantStudio5 system (Applied Biosystems). U6 was used as the internal control for the validation of miRNAs. The relative expression level was measured using the comparative 2^−ΔΔCT^ method (Livak and Schmittgen, 2001).

**TABLE 1:** miRNA primers used in this study.

miRNA	miRNA specific forward primers sequence (5′–3′)
ssc-miR-10a	TAC​CCT​GTA​GAT​CCG​AAT​TTG​T
ssc-miR-4332	TGT​CGC​GGG​GGT​GGG​CGG​GC
ssc-miR-320	AAA​AGC​TGG​GTT​GAG​AGG​GCG​AA
ssc-miR-452	AAC​TGT​TTG​CAG​AGG​AAA​CTG​A
ssc-miR-184	TGG​ACG​GAG​AAC​TGA​TAA​GGG​T
ssc-miR-451	AAA​CCG​TTA​CCA​TTA​CTG​AGT​T
U6	F: GCT​TCG​GCA​GCA​CAT​ATA​CT
	R: TTC​ACG​AAT​TTG​CGT​GTC​AT

### 2.4 Statistical analysis

All experiments were repeated at least three times, and the data were presented as mean ± standard deviation. The data were analyzed using the SPSS software version 20.0 (SPSS Inc., Chicago, IL, United States). Unpaired *t*-test was used to analyze the significance of statistics. *p*-values <0.05 were considered to be statistically significant, while *p*-values <0.01 was considered to be statistically extremely significant.

## 3 Results

### 3.1 Overview of small RNA sequencing

**FIGURE 1:**
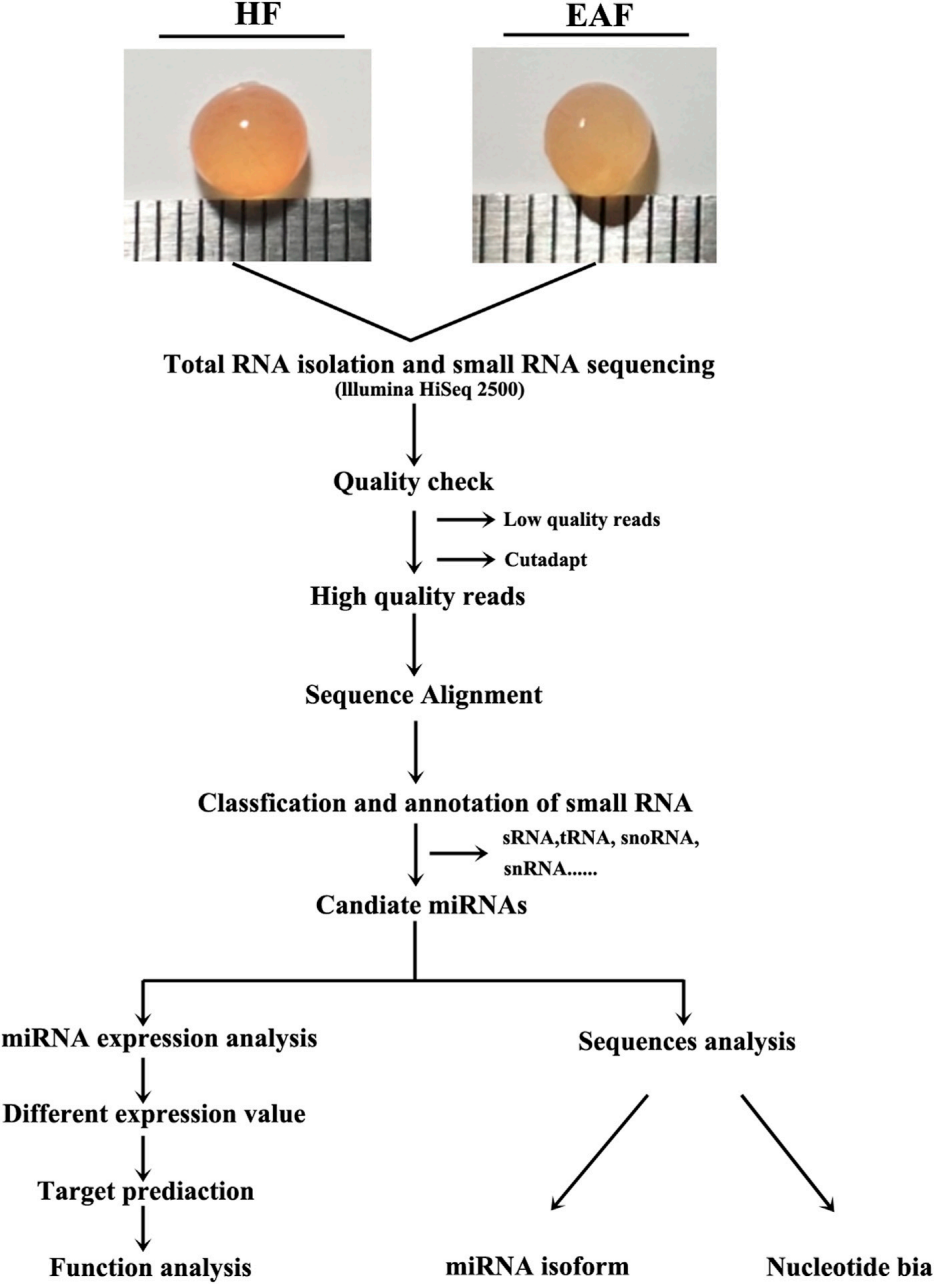
Schematic diagram of the experiment. HF, healthy follicles; EAF, early atresia follicles. HF, healthy follicle, 3–5 mm with blood vessel, clear follicular fluid and pink color; EAF, early atretic follicle, 3–5 mm with turbid follicular fluid and yellow color.

**FIGURE 2:**
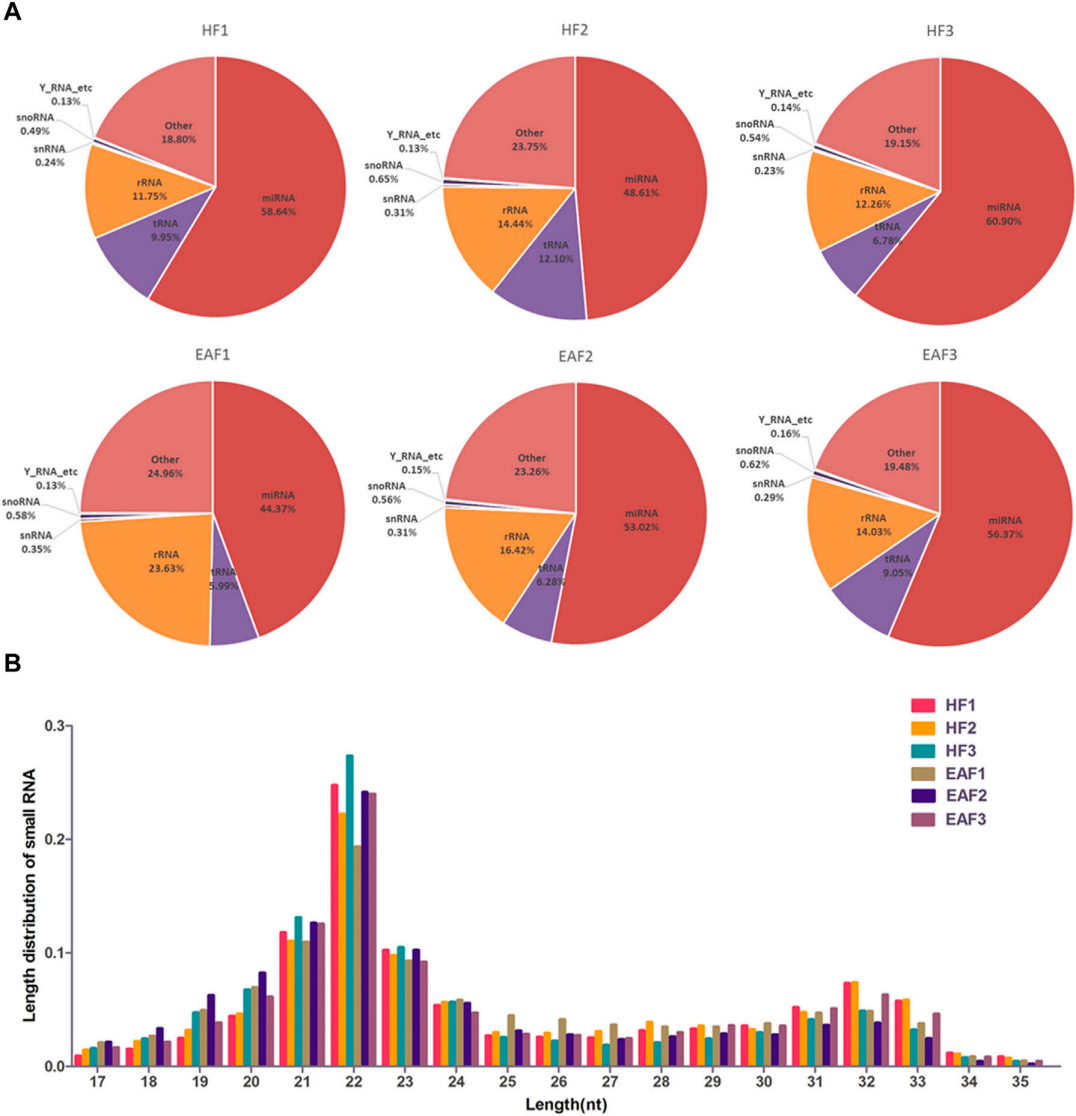
The composition analysis of small RNA and length distribution of miRNAs. **(A)** The composition analysis of small RNA; **(B)** the length distribution of miRNAs; HF, healthy follicles; EAF, early atresia follicles.

As shown in Figure 1, after the removal of low-quality reads and adaptor sequences, a total of 15,164,669, 14,443,413, 16,382,285, 15,279,119, 17,165,669 and 16,803,580 clean reads were obtained from HF1, HF2, HF3, EAF1, EAF2 and EAF3 libraries, respectively. Subsequently, the transcriptome sequences were aligned to whole pig genome data (*Sus scrofa* 11.1) from the NCBI miRNA database, and 14,862,688 (98.01%), 14,119,933 (97.76%), 16,105,126 (98.31%), 14,948,455 (97.84%), 16,874,407 (98.3%) and 16,473,089 (98.03%) small RNA reads were perfectly mapped onto the sus s*crofa* transcriptome reference sequences from healthy follicles and early atretic follicles libraries. The composition analysis of small RNA shows that the percent distribution of miRNA accounted for 58.65% in HF1, 48.61% in HF2, 60.90% in HF3, 44.37% in EAF1, 53.3% in EAF2 and 56.36% in EAF3 (Figure 2A). Meanwhile, it is observed that the numbers of small RNA with 20–24 nucleotides dominate the small RNA population, and this length section is also consistent with the feature of miRNA (Figure 2B).

### 3.2 miRNA identified

**FIGURE 3:**
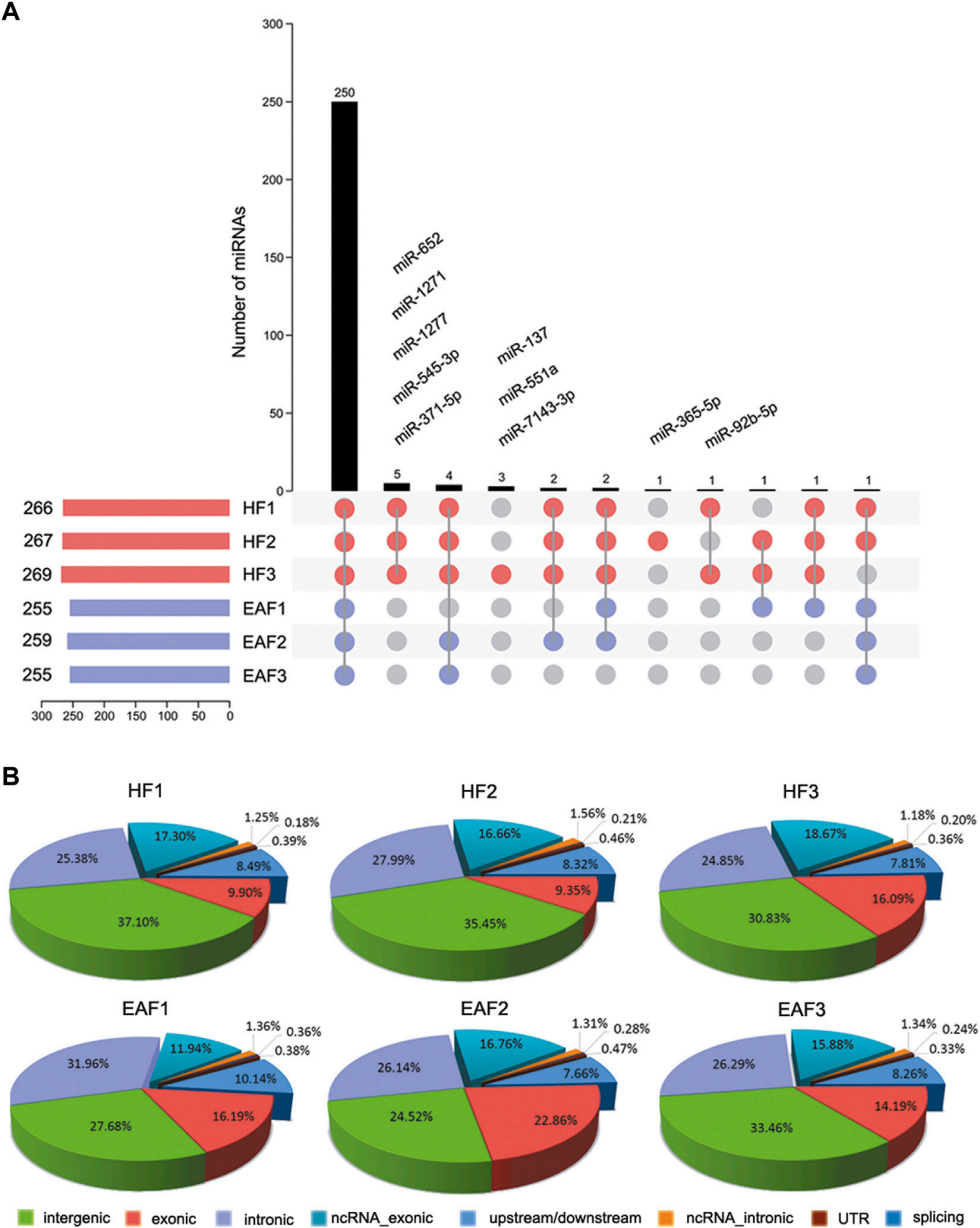
Characterization and distribution analysis of miRNAs. **(A)** Known miRNA acquisition and specific expression of miRNAs in HF; **(B)** the Percentage of mature miRNAs classified according to their position in the genome. HF, healthy follicles; EAF, early atresia follicles.

**FIGURE 4:**
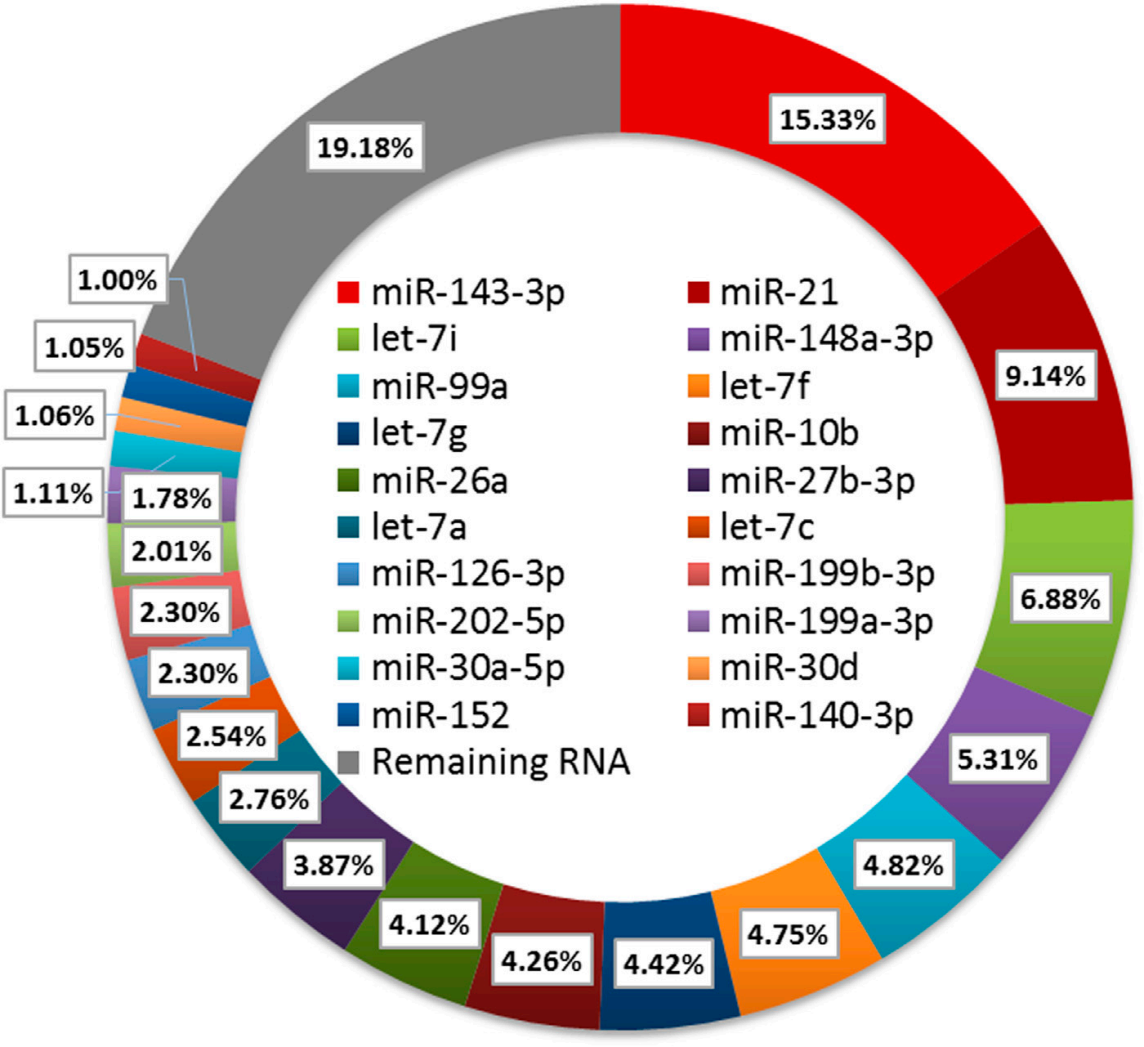
Expression analysis of the DE miRNAs.

To further characterize miRNA in ovarian follicles, the small sequences were blasted against the miRNA precursors and mature miRNAs in the miRBase database. A total of 250 known miRNAs were commonly identified in the six follicular, and 10 miRNAs (ssc-miR-371-5p, ssc-miR-652, ssc-miR-1277, ssc-miR-545-3p, ssc-miR-1271, ssc-miR-137, ssc-miR-551a, ssc-miR-7143-3p, ssc-miR-365-5p and ssc-miR-92b-5p) were explicitly expressed in the HF libraries (Figure 3A). Moreover, we classify miRNAs according to their position in the genome, and the most abundant miRNAs were located in intergenic (Average ratio: 31.51%) and intronic (Average ratio: 27.10%) (Figure 3B). Among these miRNAs, the expression distribution was very imbalanced, and only 20 miRNAs account for more than 80% of total miRNA expression (Figure 4). Compared with other miRNA expressions (Huang et al., 2016; Bick et al., 2018), ssc-miR-143-3p (15.33%), ssc-miR-21(9.14%) and ssc-miR-148 (5.31%) were found to be abundant, which was consistent with previous studies about miRNA profiling in pig (Figure 4).

### 3.3 IsomiRs analysis

**FIGURE 5:**
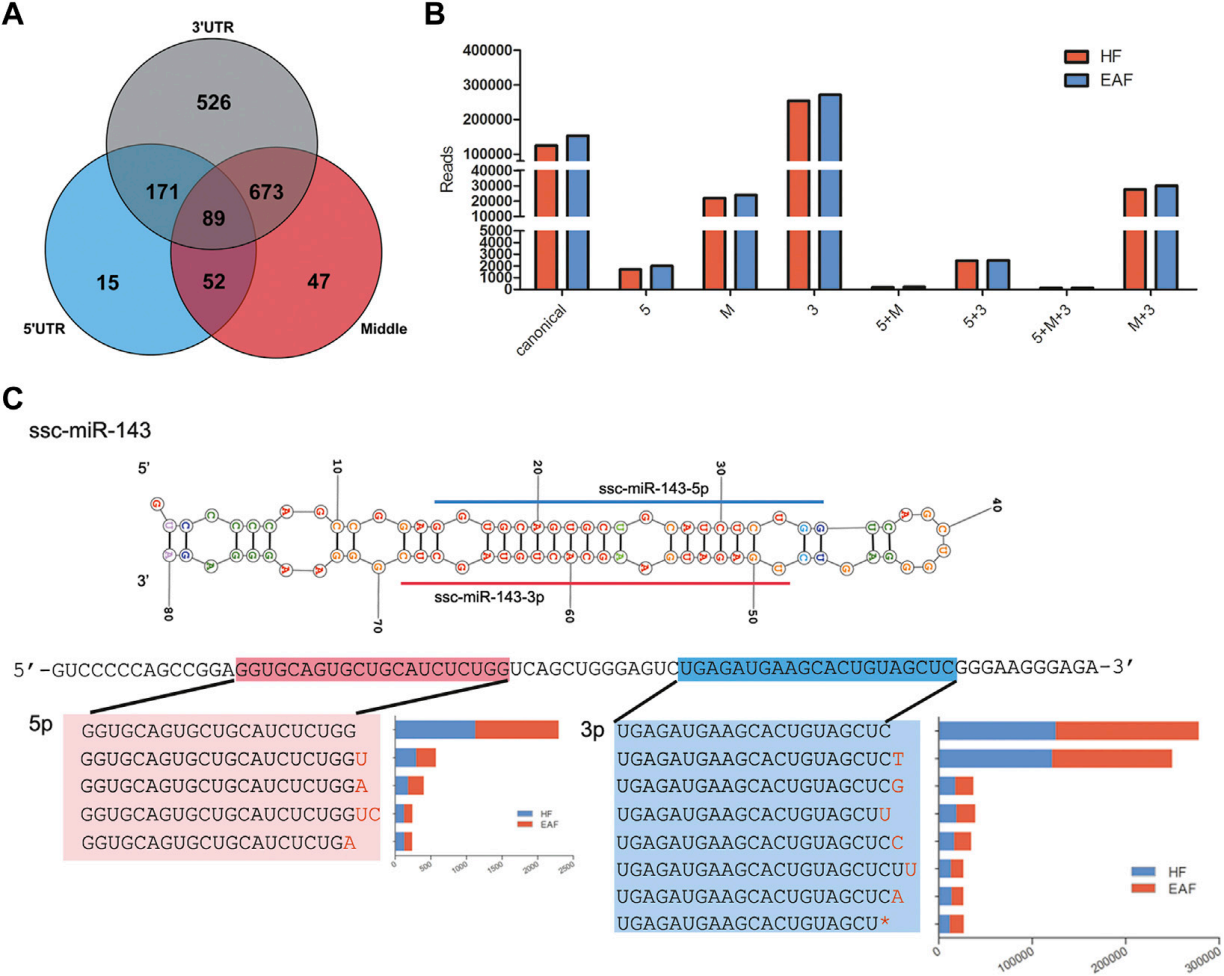
IsomiRs analysis. **(A)**, Distribution of isomiRs derived from 5′^,^ UTR (blue), 3′^,^ UTR (grey) or from the middle of the strand of a miRNA (red); **(B)**, Abundance of diverse miRNAs, including canonical miRNAs, isomiRs from 5′^,^ UTR and 3′^,^UTR; **(C)**, Details on the top 7 expressed isomiRs of ssc-miR-143-3p.

IsomiRs are a series of miRNAs variants modified by adding, editing, or subtracting nucleotides. According to the position of the mutant, the isomiRs can be classified as 5′isomiRs, 3′isomiRs, and polymorphic isomiRs. In the present study, sequence diversity was widely observed in miRNAs, and a total of 903 co-expressed isomiRs with reads not less than 100 were chosen to analyze. As shown in Figure 5A, we detected 74 variants in the 5′region, 313 variants in the internal region, and 704 variants in the 3′region (Figure 5A). We can also observe that there are 141 and 43 3′isomiRs, respectively, having variants in the internal region and 5′region, and 4 isomiRs have mutants in all three regions (Figure 5A). Among these isomiRs, the proportion of 3′end variants’ distribution and expression is the highest, and their expression pattern was the same as canonical miRNAs in HF and EAF (Figure 5B). For example, as the highest expressed miRNA in follicles, the top 7 expressed isomiRs (reads >1,000) of ssc-miR-143-3p all mutated in the 3′end, and the predominant type of 3′isomiRs were adding base rather than deletions (Figure 5C).

### 3.4 Differentially expressed miRNAs in HF and EAF

**FIGURE 6:**
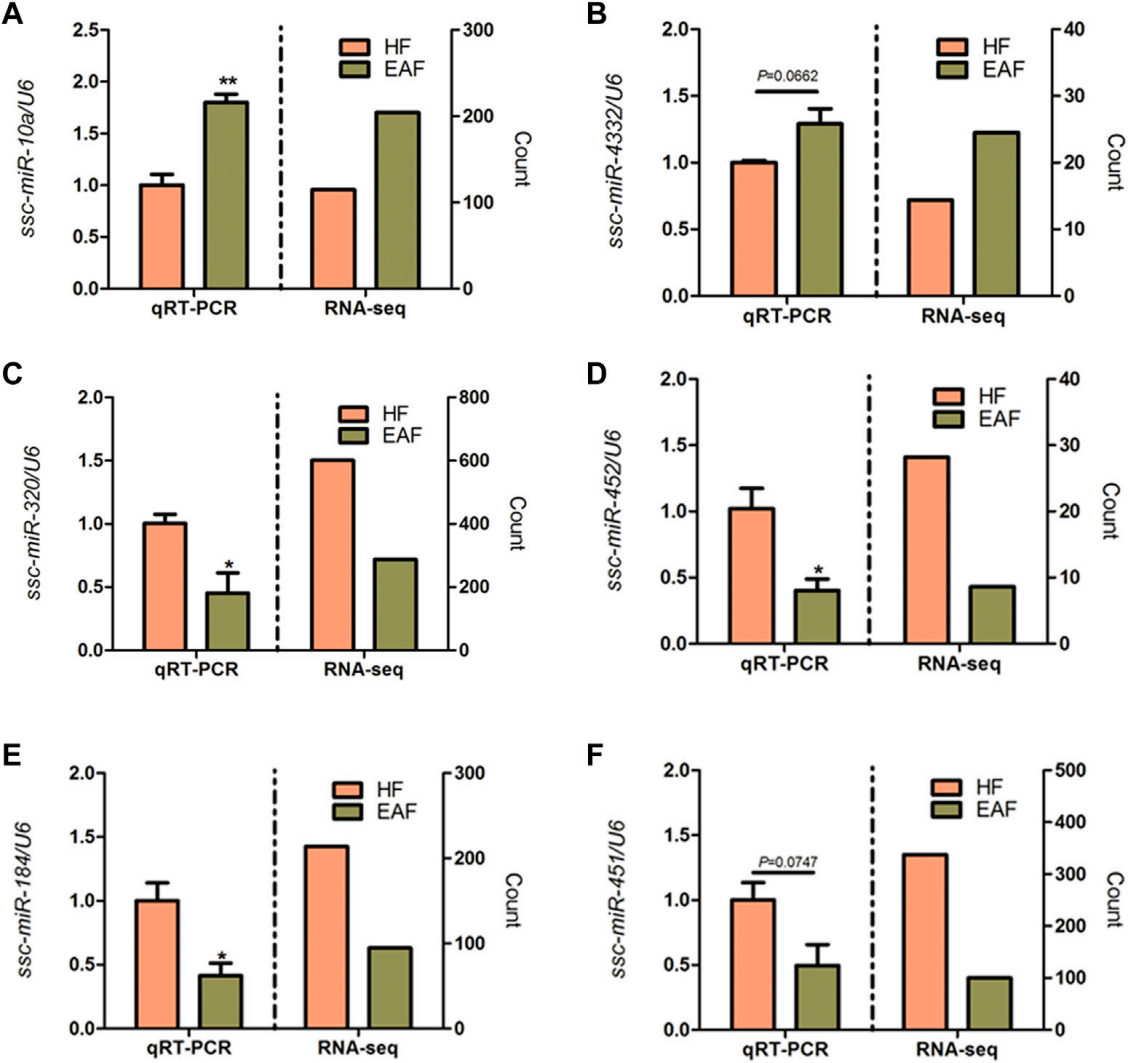
qRT-PCR validation of six DE miRNAs. *p < 0.05; **p < 0.01. Data are shown as mean ± SE. **(A–F)** represent DE miRNAs.

Read counts were analyzed by the Deseq2 algorithm to identify differentially expressed miRNA between HF group and EAF group. Filtering by |foldchange|≥1.5 and *p* < 0.05, 22 differentially expressed miRNA were detected, of which 19 were upregulated and 3 were downregulated in the EAF group compared with the HF group (Table 2). Among these differential miRNAs, the expression of ssc-miR-320, ssc-miR-423, and ssc-miR-451 were in the top 3. Meanwhile, we noted that the numbers of two miRNA clusters, miR-183–96–182 cluster and miR-144/451 cluster, were significantly downregulated in EAF collectively. It indicated that the transcriptional levels of these miRNA clusters might be regulated by the common factor in EAF. To further validate the differentially expressed miRNAs identified by RNA-seq, a total of 6 miRNAs (ssc-miR-10a, ssc-miR-4332, ssc-miR-320, ssc-miR-452, ssc-miR-184, ssc-miR-451) were selected for adding A tail qRT-PCR. We observed a good agreement between RNA-seq and qRT-PCR expression (Figure 6), and these results suggested that the expression patterns of the differentially expression miRNAs corresponded to the results detected by the real-time PCR (the *p*-values for qRT-PCR data analysis are 0.0083 for ssc-miR-10a, 0.0662 for ssc-miR-4332, 0.0371 for ssc-miR-320, 0.0429 for ssc-miR-452, 0.0437 for ssc-miR-184, and 0.0747 for ssc-miR-451 respectively).

**TABLE 2:** Details of the DE miRNAs.

miRNA_ID	Fold change	*p*-value	Regulation
ssc-miR-423-5p	−1.88	0.012	down
ssc-miR-320	−2.09	0.004	down
ssc-miR-187	−2.21	0.016	down
ssc-miR-874	−2.25	0.004	down
ssc-miR-184	−2.26	0.003	down
ssc-miR-7135-3p	−2.35	0.040	down
ssc-miR-210	−2.38	0.003	down
ssc-miR-182	−2.43	0.006	down
ssc-miR-34c	−2.51	0.001	down
ssc-miR-2366	−3.03	0.001	down
ssc-miR-452	−3.27	4.3E-07	down
ssc-miR-451	−3.38	0.001	down
ssc-miR-486	−3.65	0.001	down
ssc-miR-138	−3.74	0.006	down
ssc-miR-122	−4.73	4.35E-05	down
ssc-miR-144	−5.26	0.022	down
ssc-miR-1277	−5.87	1.37E-06	down
ssc-miR-183	−5.99	4.24E-05	down
ssc-miR-96-5p	−10.23	1.48E-05	down
ssc-miR-10a-5p	1.78	1.64E-05	up
ssc-miR-4332	1.71	4.47E-04	up
ssc-miR-7857-3p	1.66	0.011	up

### 3.5 Target gene prediction and functional annotation

**FIGURE 7:**
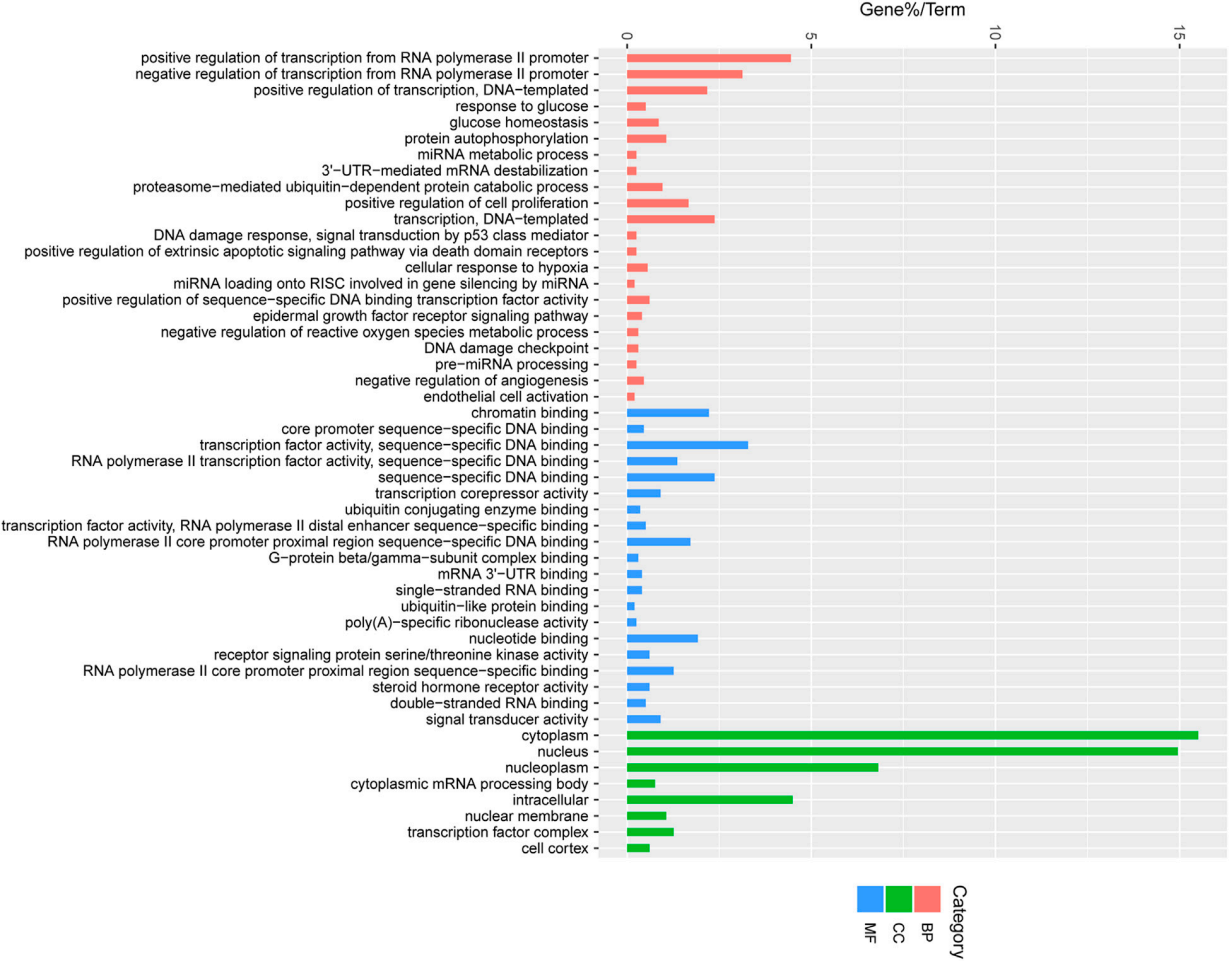
Enriched gene ontology (GO) terms of the targeted genes by the DE miRNAs between HF and EAF. HF, Healthy follicles; EAF, Early atresia follicles; *Y*-axis, the GO terms names; *X*-axis, the percent of genes enriched in this GO terms. Pink colour represents molecular functions; Green colour represents cellular components; Blue colour represents biological processes.

To better understand the function of miRNAs in the porcine follicular atresia, it is essential to identify their target genes. We used TargetScan, miRanda and TarBase to predict the target genes of the above 22 DE miRNAs. Then, all the target genes were subjected to GO and KEGG pathway enrichment analysis. A total of 50 significantly enriched GO terms were identified, including 22 under molecular function, 8 under cellular component, and 20 under biological process. We observed a cluster of terms that play potential roles in follicular atresia, such as transcription regulation, miRNA silencing, ubiquitin process, DNA damage, regulation of proliferation and apoptosis. For instance, the significantly enriched terms of biological process involved positive or negative regulation of transcription from RNA polymerase II promoter, positive regulation of cell proliferation, extrinsic apoptotic signaling pathway via death domain receptors, ubiquitin process, regulation of proliferation or apoptosis, miRNA metabolic process, and DNA damage checkpoint; The term of molecular function refer to transcription factor activity, ubiquitin-like protein binding, sequence-specific DNA binding, and steroid hormone receptor activity; For terms of cellular component, the target mainly enriched in transcription factor complex (Figure 7).

**FIGURE 8:**
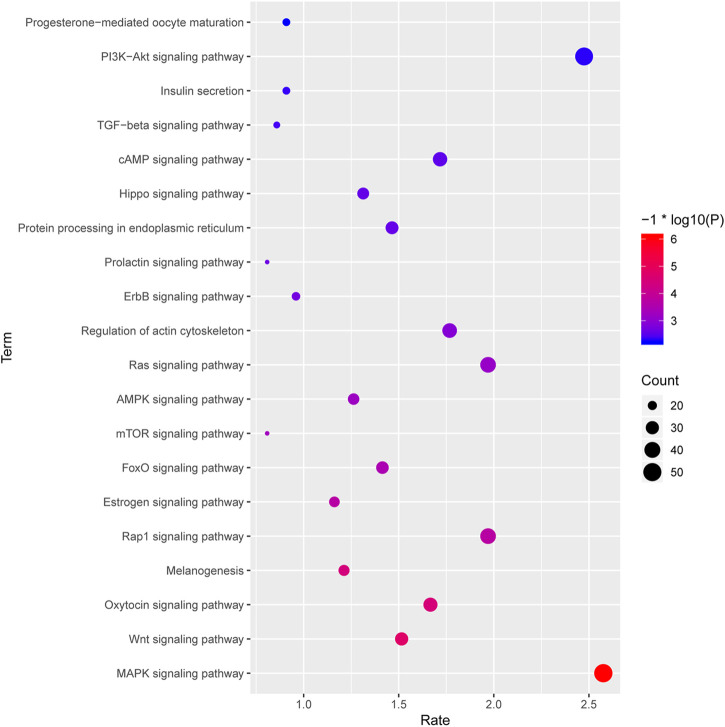
The top 20 significantly enriched pathways of the targeted genes by the DE miRNAs between HF and EAF. *X*-axis, the richness rate; *Y*-axis, the pathway name; The size of the circular, the count of genes enriched in this pathway. Only the significantly differential KEGG terms were shown here. Red, high degree of enrichment; Blue, low degree of enrichment.

Meanwhile, the top 20 significantly enriched pathways are shown in Figure 8. We can observe that MAPK signaling pathway, Wnt signaling pathway, FoxO signaling pathway, mTOR signaling pathway, AMPK signaling pathway, and TGF-β signaling pathway were involved in the significantly enriched miRNA-associated pathways, and these pathway were mediated by various transcriptional factors (Figure 8). Besides, KEGG analysis shows that the oxytocin signaling pathway, estrogen signaling pathway, and Insulin secretion are also significantly enriched, and it suggests that hormone function regulated by miRNA might contribute to follicular atresia, which were also observed in previous studies (Figure 8). Furthermore, a total of 18 target genes were significantly enriched in terms of progesterone-mediated oocyte maturation and further proved that miRNA target genes play an important role in the process of hormone-mediated follicular development (Figure 8).

**FIGURE 9:**
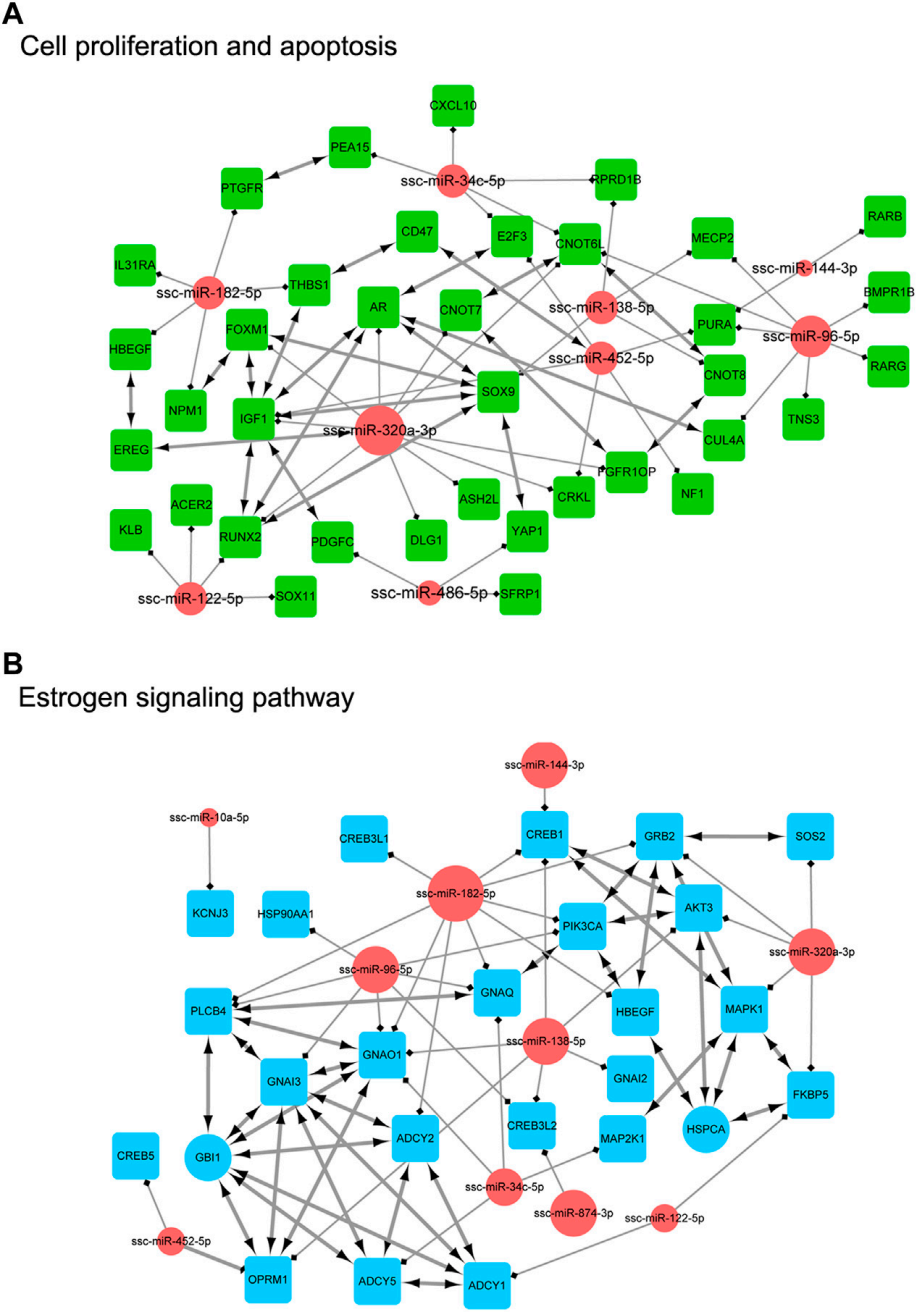
The interaction network of miRNA and their targets. **(A)** represents “cell proliferation and apoptosis”. Inside the circles are the DE miRNAs, while inside the squares are their targets. **(B)** represents “eatrogen signaling pathway”. Inside the circles are the DE miRNAs, while inside the squares are their targets.

**FIGURE 10:**
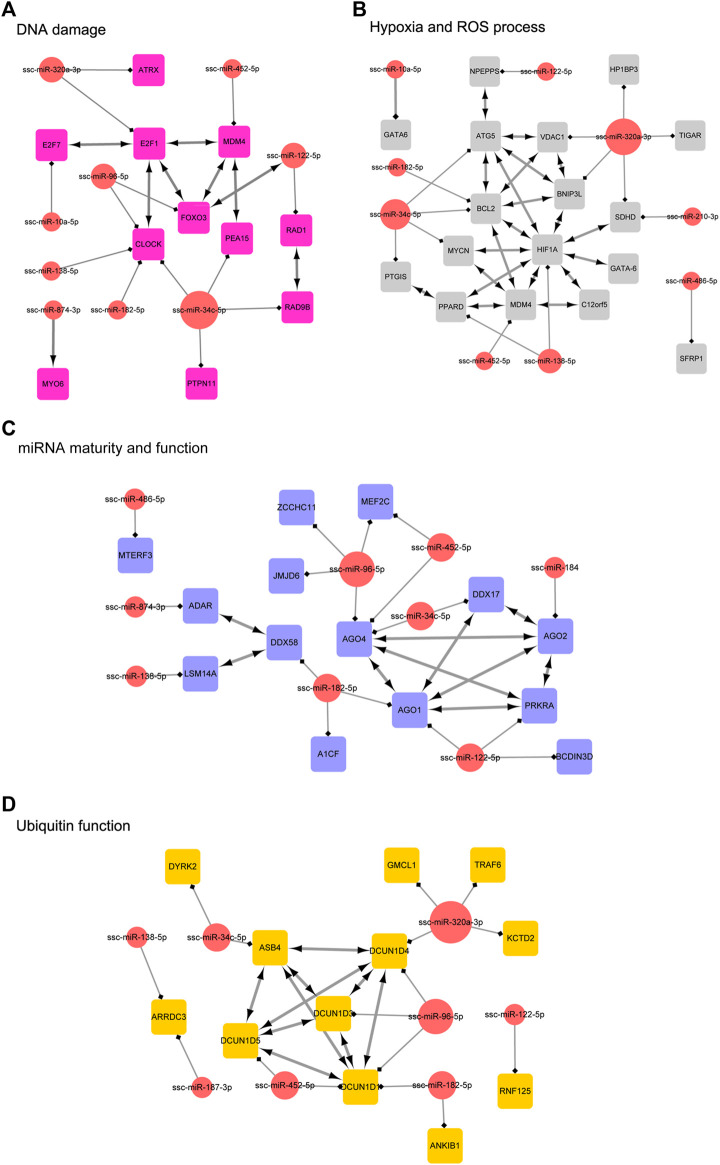
The interaction network of miRNA and their targets. **(A)** represents “DNA damage”. Inside the circles are the DE miRNAs, while inside the squares are their targets. **(B)** represents “hypoxia and ROS process”. Inside the circles are the DE miRNAs, while inside the squares are their targets. **(C)** represents “miRNA maturity and function”. Inside the circles are the DE miRNAs, while inside the squares are their targets. **(D)** represents “ubiquitin function”. Inside the circles are the DE miRNAs, while inside the squares are their targets.

In order to clarify the targeting relationship of differentially expressed miRNA in each functional cluster or pathway, we constructed the interaction network of miRNA and their targets enriched in multiple terms involving cell proliferation and apoptosis, estrogen signaling pathway, DNA damage, Hypoxia and ROS process, miRNA mature and function, and ubiquitin process (Figure 9; Figure 10). It is observed that several genes that interact in the special pathway were involved in the regulation of selected miRNA beyond single targets, suggesting their influence on pathway regulation by coordinated activity on several functionally connected genes. For example, ssc-miR-320a-3p is predicted to target various genes in cell proliferation and apoptosis process, estrogen signaling pathway and ubiquitin function (Figures 9A, B; Figure 10D); ssc-miR-96-5p and ssc-miR-34c-5p are in the center of target network of DNA damage and miRNA function respectively (Figures 10A, C); ssc-miR-138-5p has been predicted target the transcriptional factor of HIF1A which has strongly regulatory role in hypoxia (Figure 10B). These results indicate the complex regulatory role of miRNAs in different biological processes that potentially contribute to follicular atresia.

## 4 Discussion

Accumulating studies have proved miRNA were strongly related to follicular atresia, which process can limit reproductive efficiency. In the present studies, we focus on the miRNA expression profiles in the initial stage of follicular atresia, and a total of 22 differentially expressed miRNAs were discovered. Although a few similar microarray approaches have been used previously to characterize the miRNA expression profiles during follicular atresia in sow (Lin et al., 2012) or cattle (Donadeu et al., 2017), this present study first used the small RNA high-throughput sequencing technology to analyze the sequences and expression of miRNA in 3–5 mm healthy follicles and early atretic follicles. Different from microarray, RNA-Seq has the advantages of high throughput, accuracy, repeatability and low signal-to-noise ratio and can detect miRNA variants and novel miRNAs.

Although miRNAs were generally conserved among species, a few miRNAs have been confirmed to be specific to a particular species, such as human-specific miR-941 (Hu et al., 2012) and chicken-specific miR-757 (Xu et al., 2006). Meanwhile, it was known that miRNAs were expressed in specific times and tissues, and these characteristics make miRNA detection more difficult. However, the deep sequencing technology performed in different species, different tissue, or different physiological conditions made it possible to detect the novel miRNAs, and accumulating novel miRNAs were identified in the ovary of other species, such as human (Xu et al., 2016), bovine (Navakanitworakul et al., 2016), zebrafish (Zayed et al., 2019), sheep (Miao et al., 2016), duck (Yang et al., 2020) and chicken (Wu et al., 2017). For porcine miRNA detection, previous studies have identified several novel miRNAs in lung, hepatic, testes, muscles, and adipose (Wang et al., 2017a; Zhang et al., 2018b; Ford et al., 2018; Luo et al., 2018; Liu et al., 2019a). Huang et al. predicted a total of 83 novel miRNAs in the ovary of Yorkshire pigs by hairpin structure analysis after Solexa sequencing, of which 37 miRNAs might related to litter sizes (Huang et al., 2016).

### 4.1 DE isomiRNAs may play a role in follicular atresia

Recent studies have demonstrated that the isoform of individual miRNAs (isomiRNAs) has broader implications on target selection, miRNA stability, or a different loading into the RISC complex (Urgese et al., 2016). Although miRNA have been identified to be highly conserved among different species, it has proved that the miRNA variant can be generated by exoribonucleases, nucleotide transferases, RNA editing, and single nucleotide polymorphisms (SNPs) (Neilsen et al., 2012). Considering the characteristics of cell or tissue-specific isomiRs, it is meaningful to analyze the miRNA variant in ovarian follicles. Ssc-miR-143, the highest expressed miRNA in follicles, was detected to have 1,574 isomiRs. Among these isomiRs, we observed that the types and expression level of 3′isomiRs were higher than 5′isomiRs, and this finding is consistent with the isomiRs expression patterns in most animals and plants from previous studies. Most 3′miRNAs variability seems to have no change on seed sequences and target function. However, it has been identified that 3′isomiRs have a differential half-life (e.g., miR-122) and AGO loading (e.g., miR-182) preference compared with canonical miRNAs (Katoh et al., 2009; Burroughs et al., 2011). Interestingly, we found a polymorphic isomiR with “G" mutated into “C" in nucleotide 4 has high expression among all ssc-miR-143 isomiRs. The seed region polymorphisms affect the function and target of miRNAs. For example, miRNA-140 with seed region mutation targets many genes important for skeletal development and homeostasis, including Loxl3, Btg1, and Trps1, and demonstrated that binding of YBX1, an RNA binding proteins (RBP), to several miR-140-5p-G exerting target repression (Grigelioniene et al., 2019). The mutant form of miR-184 fails the competition role of wild-type miR-184 with miR-205 for targeting the 3′UTRs of INPPL1 and ITGB4 and contributes to familial keratoconus with cataracts (Hughes et al., 2011). Besides, point mutations in the seed sequence of miR-142-3p have been proven to be associated with acute myelogenous leukemia (AML), resulting in loss of miR-142-3p function and decreased miR-142-5p expression (Trissal et al., 2018).

### 4.2 MiRNA can affect follicular atresia through multiple pathways

Previous studies have demonstrated that miRNAs were involved in follicular atresia by regulating various pathways, cellular functions or biological processes. In particular, the granulosa cells’ survival or death was considered to be strongly related to follicular development or atresia. Lin et al. (2012) performed a miRNA microarray assay in healthy, early atretic. They found a total of 23 miRNAs whose targets have the function of cell proliferation, differentiation, apoptosis, or DNA replication that might be involved in the initiation of follicular atresia. In our study, we detected a total of 22 known miRNAs differentially expressed between HF and EAF. Interestingly, the function-enriched analysis and functional term classification show that these miRNAs not only regulate cell proliferation and apoptosis that were consistent with previous studies (Cao et al., 2015) but also play a role in additional biological functions that have a potential relationship with the initiation of follicular atresia, such as cell proliferation and apoptosis, estrogen signaling, DNA damage, hypoxia or ROS process and so on.

#### 4.2.1 Cell proliferation and apoptosis

The fate of granulosa cells was strongly relative to ovarian follicular development and maturity. Maintaining a good proliferation level of granulosa cells is helpful to improve the quality of follicles. Contrarily, excessive apoptosis of granulosa cells triggers follicular atresia. Consistent with previous studies, we found a total of 9 differentially expressed miRNAs were involved in cell proliferation and apoptosis. In these miRNAs, we can observe that the miR-320a-3p was predicted to target multiple genes that play essential roles in cellular fate, such as IGF1, AR and FOXM1. IGF1 has been proven to enhance cellular proliferation and reduce apoptosis during folliculogenesis. Meanwhile, some studies have suggested that the function of miR-320 targeting IGF1 was related to angiogenesis in diabetic hearts, myocardial ischemia and reperfusion injury, and brain parenchyma injury via regulating cellular proliferation or apoptosis levels (Wang et al., 2009; Song et al., 2016).

Interestingly, Huat et al. (2015) found that IGF1 also suppressed the expression of miR-320 in bone marrow mesenchymal stem cells and indicated the feedback regulation relationship between IGF1 and miR-320. AR-mediated androgen actions play a role in regulating female fertility and follicle health, development and ovulation (Walters et al., 2019). Besides, in kidney cancer, bladder cancer and prostate cancer, AR has been identified to be critical to multiple tumors’ development and progression via promoting cell proliferation and suppressing apoptosis, and especially in prostate cancer, AR activity was suppressed by miR-320a (Ding et al., 2016; Pak et al., 2019; Zheng et al., 2020), which also was observed in our target network. Similarly, a series of studies have shown that the miR-320a inhibits tumor cell proliferation by targeting FoxM1. As a member of FOX family transcription factors, FoxM1 functioning downstream of the PI3K-Akt, Ras-ERK and JNK/p38MAPK signalling cascades cooperate with FoxO3, are crucial for cell proliferation, cell survival, DNA damage repair and cell cycle control (Yao et al., 2018). Considering the various functions of the FOX family in ovarian follicles, we suspected the regulation of FoxM1 by miR-320 might contribute to follicular atresia and granulose cell fate.

#### 4.2.2 Estrogen signaling

Estrogen activation of estrogen receptors (estrogen receptor-alpha [ER-α] and estrogen receptor-beta [ER-β]) is essential for folliculogenesis via regulation of granulosa cell proliferation and apoptosis, promoting of follicular mature and ovulation, activation of primordial follicles, and controlling of angiogenesis. In our studies, we found that several differentially expressed miRNAs (ssc-miR-182-5p, ssc-miR-138, ssc-miR-425-5p,ssc-miR-34c-5p, miR-874-3p, and miR-122-5p) all can target genes from ADCY family (ADCY-1,2,5) and CREB family (CREB-1,5, 3L2, 3L1), which are respectively upstream and downstream of cAMP/PKT signaling regulation in estrogen synthesis. ADCY functions on catalyzing ATP to cyclic AMP (cAMP), and as a secondary messenger, cAMP produced activates protein kinase A, which catalyzes phosphorylation of cAMP response element binding protein (CREB) leading to activation of transcription of StAR to product estrogen (Zhao et al., 2020). In mouse granulosa cells, knockdown of CREB1 decreased the estradiol synthesis (Zhang et al., 2018c). Similarly, previous studies have demonstrated that miR-27a and miR-205 suppress CREB1, inducing the expression of Cyp19a1, a crucial enzyme of estrogen synthesis (Wang et al., 2017b; Zhang et al., 2019b). Meanwhile, ADCY2 was predicted as a target of miR-182 in stem cells and prostate cancer (Zhang et al., 2016; Zhao et al., 2020), which was also observed in our analysis. Pesiri et al. (2015) found that estrogen receptors regulate the 17β-estradiol-induced CREB activation and cell proliferation via PI3K/AKT pathway as a feedback mechanism. In mesenchymal stem cells, E2 was identified to promote ADCY2 expression by inhibiting miR-152 and miR-148a (Zhao et al., 2020). Generally, ADCY and CREB control the synthesis of estrogen and are activated by estrogen, and their regulation by miRNA might play an essential role in estrogen signaling–mediated follicular growth and atresia.

In addition, the ssc-miR-34c-5p and ssc-miR-320-3p downregulated in EAF were predicted to target the MAP2K1 and MAPK1 from the p38/MAPK signaling, which was activated by estrogen receptor *a* or *ß* (Klinge et al., 2005; Li et al., 2016; Liu et al., 2019b). As a vital protein kinases pathway, p38/MAPK is activated during the resumption of first oocyte meiosis and plays a wide range of physiological functions in the ovary, including meiotic spindle assembly, cell cycle progression, granulosa cells proliferation and apoptosis, cumulus expansion, ovulation, and corpus luteum formation (Chen et al., 2020). During the follicular development, the phosphorylated MAPK expression showed an upward trend and presented low abundance in granulosa cells of atretic follicles lacking FSH (Ilha et al., 2015). Recent studies have proved that MAPK signaling is essential for EGFR-induced E2 production, GCs proliferation, and follicular development (Wu et al., 2019). In mouse granulosa cells, p38-MAPK-mediated dephosphorylation of STAT1 downregulated cyp1b1 to maintain the estradiol levels in dominant follicles (Du et al., 2015). Previous studies have identified that the miR-34c and the miR-320 act as regulatory molecules of insulin-producing in mesenchymal stem cells or cell proliferation in ovarian tumor cells via targeting to MAPK1 or MAP2K1, respectively (Bai et al., 2017; Xu et al., 2017). Summarily, we speculated that the expression change of ssc-miR-34c-5p and ssc-miR-320-3p might affect the activation of p38/MAPK signaling, and then leading to an imbalance of estradiol synthesis and signal mediation, which was relative to follicular atresia.

#### 4.2.3 Accumulation of DNA damage in follicles inhibits the activation of primitive follicles

DNA damage accumulation in follicles facilitates the suppression of primordial follicle activation, blocking oocyte meiotic maturation, mediating oocyte or granulosa cell apoptosis, and promoting ovarian age (Kerr et al., 2012; Zhang et al., 2014; Maidarti et al., 2020). We observed that the targets of DE miRNAs were enriched in p53–mediated DNA damage response signal transduction. For example, ssc-miR-96-5p and ssc-miR-122-5p target to FoxO3, and miR-452-5p target to MDM4. p53 was a transcriptional regulatory protein stabilized by DNA damage response and involved in cell cycle arrest, DNA repair, and cell fate regulation. In order to activate p53, FOXO3 interacts with the ATM-Chk2-p53 complex, augments phosphorylation of the complex, and induces the formation of nuclear foci in cells on DNA damage (Chung et al., 2012). Moreover, MDM4 is a cytoplasmic protein that functions on p53-activating under DNA damage conditions by phosphorylation of p53 at Ser46, and promotes p53-mediated transcriptional repression (Mancini et al., 2016). Under DNA damage, the downregulation of ssc-miR-96-5p, ssc-miR-122-5p and miR-452-5p inducing FoxO3 and MDM4 promotes the phosphorylation of p53 which can mediate activation of apoptotic pathway (Fas/Fasl system or BCL2 family) in granulosa cells or oocytes, and it might provide a new mechanism in initiation of follicular atresia.

Recently, it has become apparent that the circadian clock plays a vital role in determining the strengths of cellular responses to DNA damage (Sancar et al., 2010). The Clock, as a crucial transcriptional activator in clock, was predicted to be targeted by various miRNAs differentially expressed in EAF compared with HF, including ssc-miR-96-5p, ssc-miR-138-5p, ssc-miR-182-5p, and ssc-miR-34c-5p. The heterodimer forming by Clock with BMall can activate transcription of Cry and Per genes, which generate a transcription-translation feedback loop, or facilitate rhythmic expression of clock-controlled genes, and current evidence indicates that these processes were also involved in DNA damage response such as DNA repair, cellular cycle checkpoints, and apoptosis (Sancar et al., 2010). In addition, the ovary is a reproductive organ with elegant and precise rhythmicity, and the Circadian Clock has been found to function in theca cells, granulosa cells, and oocytes to affect the processes of follicular growth, steroid hormone synthesis, and ovulation (Nagao et al., 2019). Although it was still unknown whether the regulation of Clock on ovarian functions was mediated by DNA damage response, we proved a new approach for two signaling systems interface regulated by miRNAs in atretic follicles.

Under DNA damage and depletion, the expression of Clock in a human glioma cell line increased apoptosis and cell cycle arrest by down-regulating c-Myc and Cyclin B1, and upregulating p53 (Wang et al., 2016). However, in keratinocytes, silencing of Clock leads to suppression of UVB-stimulated apoptotic responses and downregulation of expression of DNA damage marker γ-H2AX and cell cycle inhibitor p21 (Sun et al., 2018).

In addition, we also observed that the downregulated miRNAs in EAF, including miR-122-5p, miR-34c-5p and ssc-miR-320a-3p, were predicted to target RAD1, RAD9B and E2F1 respectively, which were essential for DNA damage repair (Sarangi et al., 2014; Seol et al., 2018; Bigot et al., 2019; Manickavinayaham et al., 2019). Several studies demonstrate that RAD1-RAD9-HUS1 act as a damage sensor clamp to recruit DNA polymerase beta or TopBP1 to lesion sites, enabling subsequent gap filling and ligation (Toueille et al., 2004; Ohashi et al., 2014). Similarly, the acetylation of E1F2 enhance the recruitment of p300 and CBP to DNA double break and the accumulation of repair factors such as Tip60, BRG1 and NBS1 (Manickavinayaham et al., 2019). In early atretic follicles, decreasing miRNAs expression might result in the DNA damage repair activating, indicating follicles were still actively repairing the damaged DNA at the initial stage of atresia. Furthermore, we can speculate that the balance between DNA damage and repair, or regulatory miRNAs expression levels of both sides, might determine the degree of follicular atresia.

#### 4.2.4 The effect of hypoxia on follicular development

One of the characteristics of follicular atresia is the decrease of blood vessels, which means that the follicles will be in hypoxia for a long time. Accumulating evidence proved that hypoxia adversely impacts ovarian function, generating reactive oxygen species (ROS), promoting granulose cell apoptosis, suppressing follicular development, and reducing luteal growth (Lim et al., 2021). Previous studies have also demonstrated that sustained hypoxia contributes to activating autophagy or apoptosis pathways in granulose cells, contributing to follicular atresia. In the present study, we can observe that the miR-138-5p has a potential role of targeting HIF1A, which regulates numerous genes in cellular hypoxia response. In mouse and porcine granulose cells, it has been suggested that FSH-mediated HIF1A expression suppresses the hypoxia-induced apoptosis via activating mitophagy through the PINK1-Parkin pathway (Zhou et al., 2017; Li et al., 2020b). Besides, blocking of the HIF1A-VEGF pathway was shown to trigger atresia in large follicles.

Besides, as a crucial transcriptional activator of oxygen homeostasis, HIF1A regulates various downstream target genes involved in hypoxia response, some of which also were predicted as targets of downregulated miRNAs in EAF, for example, the BCL2 targeted by ssc-miR-182-5p and ssc-miR-34c, BNIP3L targeted by ssc-miR-320-3p, and ATG5 targeted by ssc-miR-34c. BCL2 interacting protein 3-like (BNIP3L/NIX) is a Bcl-2 family member with BH3 domain whose expression was dependent on HIF1A (Tang et al., 2019). Although BNIP3L was first proved as a pro-apoptosis protein, recent studies have shown that BNIP3L is essential for mitophagy recruitment of autophagosomes using its LC3 interacting region (LIR) and plays pro-survival roles in hypoxia (Esteban-Martinez and Boya, 2018; Tang et al., 2019). Similarly, previous studies have demonstrated that the phosphorylation of ATG5 at T101 enhanced the affinity of ATG12 -ATG5 -ATG16L1 complex, which drives the LC3-lipid conjugation and autophagic membrane elongation to activate the autophagy in hypoxia (Feng et al., 2021). In ovarian follicles, hypoxia-induced autophagy might play a dual role in follicular development and atresia. At the mild or initial phase of hypoxia response, autophagy, especially mitophagy induced by signaling cascade such as FSH/HIFA pathways, has been identified to sustain the survival of cells via removing damaged mitochondria, which drive apoptotic factors released. However, under extreme stress, excessive autophagy may induce granulosa cell apoptosis and follicular atresia through mediating acetylation of FOXO1 to interact with ATG proteins (Shen et al., 2018). In the present study, the miR-138-5p, ssc-miR-182-5p, ssc-miR-34c and ssc-miR-320-3p, which target various hypoxia-induced autophagy-related genes, were observed downregulated in early atretic follicles and indicated that the activation of hypoxia-induced autophagy determines the follicular fates by complex regulatory mechanism.

## 5 Conclusion

In conclusion, this study was performed to identify miRNAs involved in the follicular atresia initiation in porcine ovaries using miRNA-Seq approach. The results showed a number of key miRNAs (ssc-miR-320, ssc-miR-423, ssc-miR-451, miR-183–96–182 cluster, miR-144/451 cluster) and their target genes (IGF1, FOXM1, ADCY-1,2,5, MAP2K1, MAPK1, FoxO3, MDM4, p53, BCL2, BNIP3L, ATG5) enriched in cell proliferation and apoptosis, estrogen signaling pathway, DNA damage, Hypoxia and ROS process, miRNA mature and function, and ubiquitin process pathways. These miRNAs and their target genes may play important roles in the regulation of ovarian follicular development and are worthy of further investigation in future studies on reproductive regulation in mammals.

## Data Availability

The data presented in the study are deposited in the National Center for Biotechnology Information (NCBI) Sequence Read Archive (SRA). The accession number is PRJNA 1049127 (http://www.ncbi.nlm.nih.gov/bioproject/1049127).

## Abbreviations

COC, cumulus-oocyte complexes; GC, granulosa cell; miRNAs, MicroRNAs; GO, gene Ontology; KEGG, kyoto encyclopedia of genes and genomes pathway-based enrichment analysis; cDNA, complementary DNA; 5′UTR, 5’untranslated region; 3′UTR, 3’un-translated region; DE, differentially expressed; ROS, reactive oxygen species; FSH, follicle stimulating hormone; LH, luteinizing hormone.
